# Functions and clinical implications of the liver microenvironment in hepatic uveal melanoma metastases

**DOI:** 10.1007/s10555-025-10298-8

**Published:** 2025-10-29

**Authors:** Camille J. Cunanan, Alyssa B. Sanders, Kayla C. Gallant, Rino S. Seedor, Edward J. Hartsough

**Affiliations:** 1 Department of Pharmacology and Physiology, Drexel University College of Medicine, Philadelphia, PA 19102 USA; 2 Department of Medical Oncology, Thomas Jefferson University, Philadelphia, PA 19107 USA; 3 Sidney Kimmel Comprehensive Cancer Center, Philadelphia, PA 19107 USA

**Keywords:** Uveal melanoma, Metastatic uveal melanoma, Liver microenvironment

## Abstract

Uveal melanoma (UM) is an aggressive intraocular malignancy in adults arising from the melanocytes of the uveal tract. While primary UM lesions can be successfully treated, ~ 50% of UM patients develop metastases primarily in the liver. Patients with liver metastatic UM (LMUM) have poor prognosis and few therapeutic options. LMUM lesions are unresponsive to standard chemotherapies, targeted therapies, and immune checkpoint inhibitors — an effect at least in part associated with the detoxification function of the liver and the diverse hepatic immunological landscape. Here, we recount the etiology and molecular mechanisms in the development of LMUM, examine the influences of the hepatic tumor microenvironment (TME) on UM liver tropism, and review how the innate and peripheral immune response contributes to LMUM progression and therapeutic efficacy. The unique immunological properties of the liver coupled with the distinct growth patterns of LMUM lesions present significant challenges for developing effective treatments that can overcome this specialized microenvironment. Improved understanding of the interplay between the liver and LMUM is essential for the development of more effective diagnostic techniques and improved therapeutic outcomes.

## Uveal melanoma

**Fig. 1:**
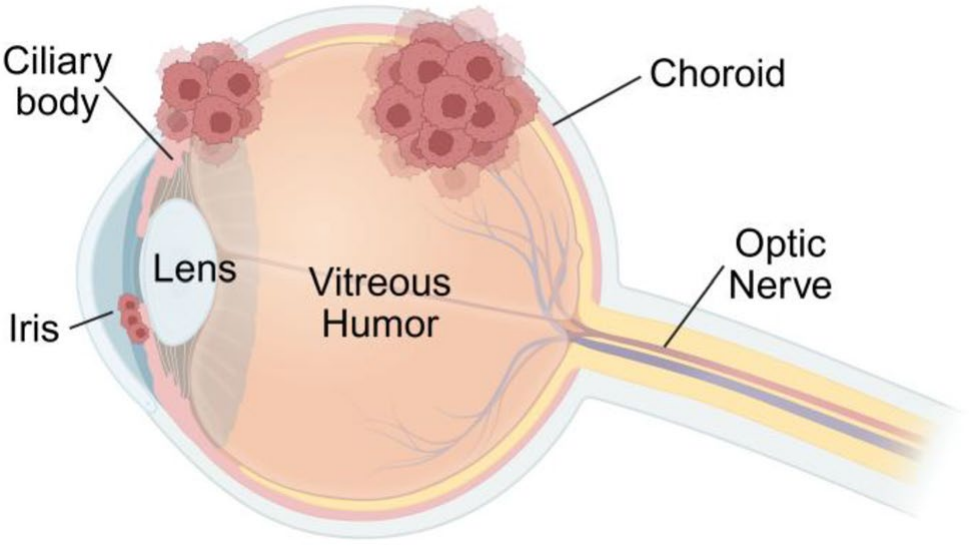
Sites of UM lesions within the eye. Cross-sectional illustration of the eye showing the locations of uveal melanoma development. UM occurs in the melanocytes of the uveal tract. The uveal tract consists of the choroid (a blood vessel-rich layer beneath the retina), the iris (the colored ring that regulates light entry), and the ciliary body (a structure that helps with lens focusing and fluid production). 90% of primary UM lesions occur within the choroid, 6% within the ciliary body, and 4% within the iris. The diagram also depicts other key ocular structures, including the lens, vitreous humor, and optic nerve

Uveal melanoma (UM) is the most common intraocular neoplasm in adults and occurs in the melanocytes of the uveal tract, which consists of the iris, ciliary body, and choroid (Fig. 1). Approximately 90% of primary UM lesions occur within the choroid, while 6% occur in the ciliary body and 4% in the iris [1–3]. UM is considered a rare disease as it comprises 3–5% of all melanoma cases and exhibits an incidence rate of 5.1 cases per million annually in the United States [4, 5]. There is a 30% greater incidence in males compared to females and a higher occurrence in non-Hispanic whites than Hispanics, Blacks, and Asians [3, 6]. Presentation of UM in children is rare, as the average age of diagnosis falls between 60 and 75 years of age, with risk factors including fair skin, blond hair, and light eye color, and a genetic predisposition to cutaneous or uveal melanoma [2]. The most common symptoms of primary UM are blurred or distorted vision, visual field loss, and changes in iris color. However, many patients are asymptomatic, which is unfortunately associated with undiagnosed disease progression — this underscores the importance of routine comprehensive eye examinations for prompt detection of UM [1, 7].

Unlike cutaneous melanoma, primary UM has a low mutational burden and is not associated with a UV damage signature [8]. Mutually exclusive mutations in G Protein Subunit Alpha Q (*GNAQ*) and G Protein Subunit Alpha 11 (*GNA11*), members of the Gαq family of proteins, frequently initiate neoplastic proliferation of uveal melanocytes, serving as a primary driver in uveal melanoma pathogenesis. Mutations in Gαq/11 predominantly occur at residues Q209 and R183 within the GTPase domain [9–11]. These mutations impair GTPase activity, resulting in constitutive activation of the Gαq signaling cascade [9–11]. This persistent activation leads to upregulation of multiple mitogenic pathways, including MAPK, YAP/TAZ, and RhoA signaling, ultimately promoting uncontrolled cell proliferation and contributing to oncogenesis [9–11]. Fortunately, over 90% of primary UM lesions can be locally controlled by radiotherapy and enucleation [4]. However, while the primary tumor can be successfully treated, over 50% of UM patients develop metastases, which confers a very poor prognosis [12, 13]. UM metastasis is often associated with secondary mutations, which separate patients into distinctly different prognoses. The Cancer Genome Atlas (TCGA) classified UM into 4 classes based on the presence and absence of chromosome 3 disomy and presence and degree of chromosome 8q gain [14]. Robertson et al. then further characterized this TCGA dataset into 4 molecularly distinct, clinically relevant subtypes [15, 16]. Group A UM patients are classified by having disomy 3, disomy 8, *EIF1AX* mutation, and favorable survival. Group B patients have disomy 3, gain of 8q, *SF3B1/SRSF2* mutations, and moderate metastatic potential. Group C is classified by monosomy 3, loss of *BAP1*, and ~33% of patients developing metastasis by 5 years. Finally, Group D is determined by monosomy 3, multiple 8q gains, loss of *BAP1*, and 5-year estimated risk of distant metastasis of ~63% [14, 15]. M3 subtypes clearly have a higher risk of metastasis and poorer prognosis. Interestingly, mesenchymal-epithelial transition factor (Met) has increased expression in a M3-UM and thus may be linked to metastasis [17]. UM can metastasize to the lungs, bone, and skin; however, 95% of patients develop metastatic UM (MUM) in the liver [13, 18].

## The liver

Nearly 50% of UM patients will develop metastatic disease, most commonly in the liver [12, 13]. It is critical to understand the liver’s structure, function, and cellular composition in order to elucidate the mechanisms underlying the liver tropism of *BAP1* mutant UM and to inform more effective therapeutic strategies.

The liver is crucial for numerous physiological processes. It is the central organ for intermediary metabolism of macronutrients, such as glucose, fatty acids, and proteins, and serves as a “biofilter” processing deoxygenated blood from the gut [19]. This gut-liver axis is a bidirectional loop — as endogenous wastes and exogenous xenobiotics, such as pathogens, drugs, and alcohol from the gut are filtered out of circulation, the liver can provide “feedback” to the gut in the form of secreted bile acids which can modulate gut function by solubilizing fats and vitamins, affecting the intestinal microbiome [20, 21]. Given the liver’s efficiency in filtering/oxidizing xenobiotics from the blood, systemic administration of pharmacological agents such as chemotherapies and targeted inhibitors can result in hepatotoxicity (reviewed in [22]). Along these lines, a successful therapeutic would need to escape (or be intentionally “activated” by) the powerful detoxifying abilities of the liver [23]. If not properly considered, these properties can lead to poor pharmacokinetics and ultimately limit the development of novel drug candidates.

**Fig. 2:**
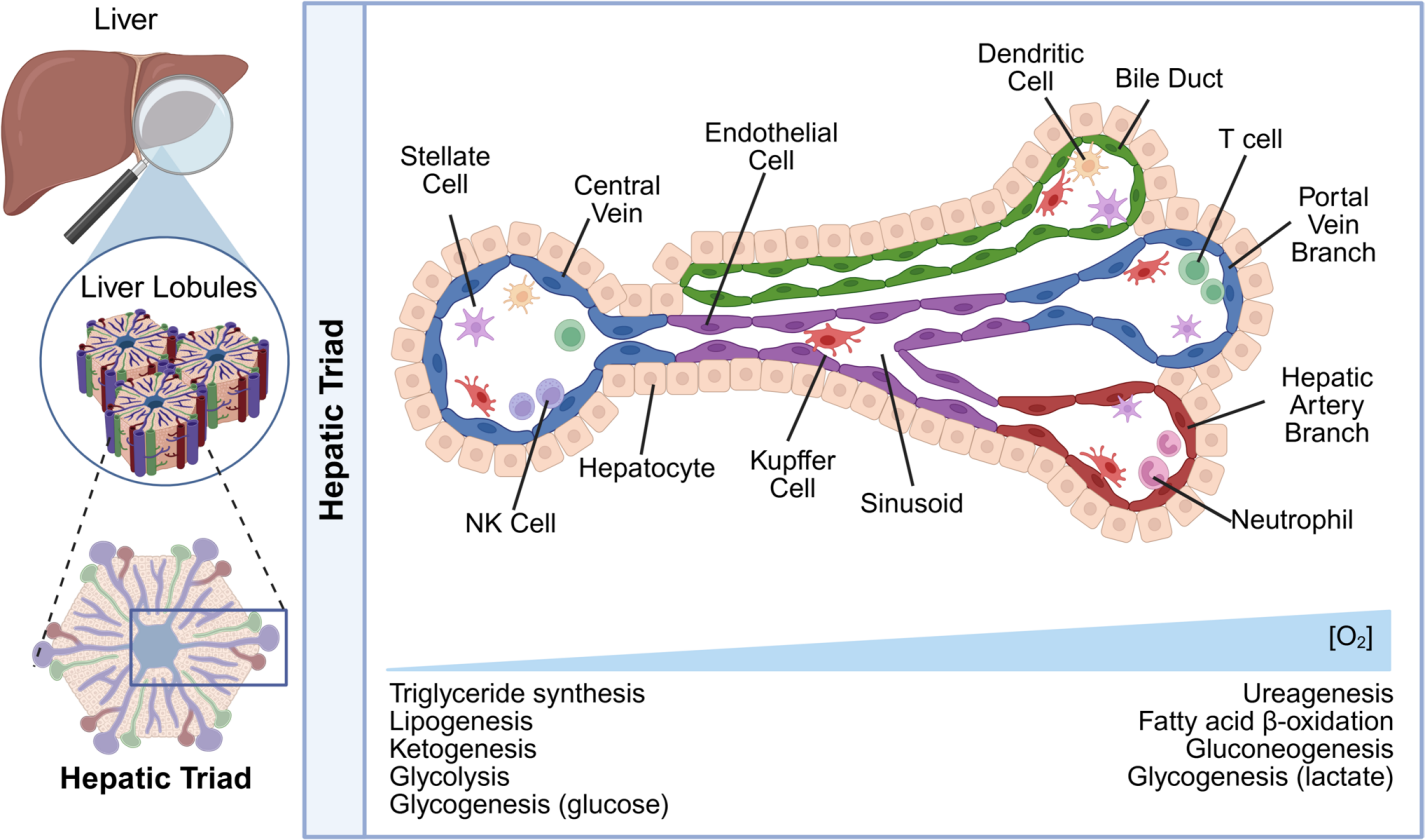
Spatial organization and cellular composition of the liver microenvironment. Hierarchical representation of liver architecture from organ to cellular level, illustrating the structural and functional units. The hepatic lobule constitutes the basic functional unit organized around the central vein. The hepatic triad consists of portal vein branch, hepatic artery branch, and bile duct. The detailed lobular structure reveals the diverse cellular composition including hepatocytes, Kupffer cells, dendritic cells, NK cells, neutrophils, T cells, stellate cells, and endothelial cells lining the sinusoids. The oxygen gradient across the lobule regulates zonation of metabolic functions, with low oxygen zones supporting triglyceride synthesis, lipogenesis, ketogenesis, glycolysis, and glycogenesis from glucose, while high oxygen zones facilitate ureagenesis, fatty acid β-oxidation, gluconeogenesis, and glycogenesis from lactate

The liver is composed of various cell types that are spatially organized around the vasculature to form a functional unit referred to as the hepatic lobule (Fig. 2). The lobule is zonated based on access to oxygenated blood stemming from the hepatic artery [19, 24]. As the blood diffuses through the lobule, an oxygen gradient forms with the lowest oxygen concentration occupying the space around the central vein, by which de-oxygenated blood is drained. Access to oxygenation dictates the metabolic functions that occur within each zone [19, 24, 25]. While ureagenesis, fatty acid beta-oxidation, gluconeogenesis, and glycogenesis from lactate occur in high-oxygen zones of the lobule, triglyceride synthesis, lipogenesis, ketogenesis, glycolysis, and glycogenesis from glucose occur in low-oxygen zones [19, 24, 25]. While the significance of liver zonation in MUM is understudied, it has been implicated in the signaling and progression of hepatocellular carcinoma (HCC). For example, single-cell RNA-seq of HCC samples illustrates that loss of zonation is associated with high-grade lesions and disease progression [26]. Given these observations in HCC, the oxygen gradient across hepatic zones might differentially influence the growth patterns of liver metastatic uveal melanoma (LMUM) lesions; potentially explaining why nodular lesions exhibiting angiogenic properties predominate in certain regions while infiltrative patterns emerge in others, though direct evidence for this relationship in LMUM has yet to be established.

The human liver primarily consists of hepatocytes, accounting for ~ 85% of the liver mass [27, 28]. Hepatocytes contribute to most metabolic functions of the liver, such as carbohydrate, protein, lipid, porphyrin, hormone, and xenobiotic metabolism [27, 28]. Hepatocytes are organized along vascular channels known as sinusoids, which receive deoxygenated portal venous and oxygenated hepatic arterial blood and flow towards the central vein. Hepatocyte membranes are composed of various surface proteins that are involved in lipid- and iron-scavenging, such as low-density lipoprotein receptor (LDLR) and transferrin receptor (TfR) [28]. Additionally, hepatocytes play a fundamental role in protein and biliary secretion, specifically the secretion of albumin, transferrin, plasminogen, fibrinogen, and other clotting factors [28]. Notably, during metastatic dissemination of pancreatic and colorectal cancer, hepatocytes undergo altered signaling mechanisms, including activation of STAT3, that may promote hepatic metastases by creating a favorable “metastatic niche” to facilitate metastatic colonization [29]. Furthermore, hepatocytes can support a favorable “metastatic niche’” through their inherent expression of CXCL12, a ligand for the receptor CXCR4, expressed on UM cells [30]. Expression of CXCL12 on hepatocytes may aid in the homing of UM cells to the liver microenvironment.

Non-parenchymal cells of the liver, including liver sinusoidal endothelial cells (LSECs), cholangiocytes, and hepatic stellate cells, play essential roles in modulating the liver microenvironment during metastasis. LSECs, which line the hepatic sinusoids, are key regulators of immune cell infiltration and interact directly with circulating tumor cells (CTCs) during the early stages of liver colonization [31]. Comparative analysis between primary UM and LMUM has revealed an enrichment of endothelial cells within LMUM tumors [32]. These endothelial cells exhibit upregulation of senescence-associated genes, such as P16 and P21, and secrete factors associated with the senescence-associated secretory phenotype (SASP), which have been shown to promote tumor cell proliferation and migration [32, 33]. Notably, BAP1 loss in UM is linked to increased expression of cell adhesion molecules and enhanced transendothelial migration [34, 35]. In colorectal cancer models, tumor-endothelial interactions via adhesion molecules facilitate liver metastasis [36]; a similar mechanism may contribute to liver tropism in UM, particularly in the context of BAP1-deficient tumors exhibiting elevated adhesion molecule expression.

Cholangiocytes are epithelial cells that line the bile duct and are involved in the secretion of water, bicarbonate, and cations into the bile [37]. Unlike other types of epithelial cells, cholangiocytes can express MHC class I and II molecules and act as APCs [38]. Cholangiocytes can become activated upon liver injury or disease and secrete cytokines and chemokines termed cholangiokines [39]. The cholangiokines secreted by cholangiocytes include, but are not limited to, TNF-alpha, IL-6, and TGF-beta [39]. In UM, upregulation of IL-6/STAT3 signaling correlated with poor survival probability [40]. Furthermore, UM cells exposed to IL-6 upregulated the expression of immune evasion proteins, PD-L1 and HLA-E [41]. Together, these results indicate that cholangiokines may aid in LMUM growth and immune invasion.

Hepatic stellate cells (HSCs) are key non-parenchymal cells that contribute to the liver’s immunoregulatory functions [42]. In response to liver injury, HSCs undergo activation and differentiate into myofibroblast-like cells, contributing to fibrogenesis through the secretion of extracellular matrix proteins and promoting a pro-tumorigenic microenvironment, including the development of hepatocellular carcinoma [43]. In the context of LMUM, HSCs appear to play a pivotal role in tumor progression. Histological analysis of LMUM patient samples revealed activated HSCs surrounding metastatic lesions, indicating their involvement in shaping the tumor microenvironment [44]. Notably, HSCs can secrete hepatocyte growth factor (HGF), which, upon binding to its cognate receptor, MET, expressed on LMUM cells, can stimulate proliferation, cell migration, and direct cell adhesion programming [17, 45, 46]. This paracrine crosstalk may support the seeding and growth of UM cells in the liver. *In vivo*, co-injection of human HSCs with UM cells into immunodeficient mice significantly enhanced hepatic metastasis compared to UM cells alone, underscoring the supportive role of HSCs in facilitating tumor seeding and outgrowth within the liver [44]. Mechanistically, HSCs engage in bidirectional crosstalk with UM cells, leading to upregulation of inflammatory cytokines (e.g., interleukins) and transmembrane integrins in tumor cells, suggesting that inflammatory signaling pathways are central to LMUM progression [47]. Collectively, these findings position HSCs as critical facilitators of the hepatic metastatic niche and highlight inflammatory signaling as a potential therapeutic target in BAP1-mutant UM.

## Uveal melanoma liver metastases

Dissemination of UM into the liver occurs as single cells that seed within the sinusoidal or periportal spaces [28]. The initial site of metastatic cell seeding influences the distinct growth patterns observed in LMUM. These growth patterns can be categorized by radiologic imaging based on the number and size of metastatic lesions. Alternatively, histopathological growth patterns (HGPs) offer insight into how LMUM cells interact with the liver microenvironment and its architecture.

**Fig. 3:**
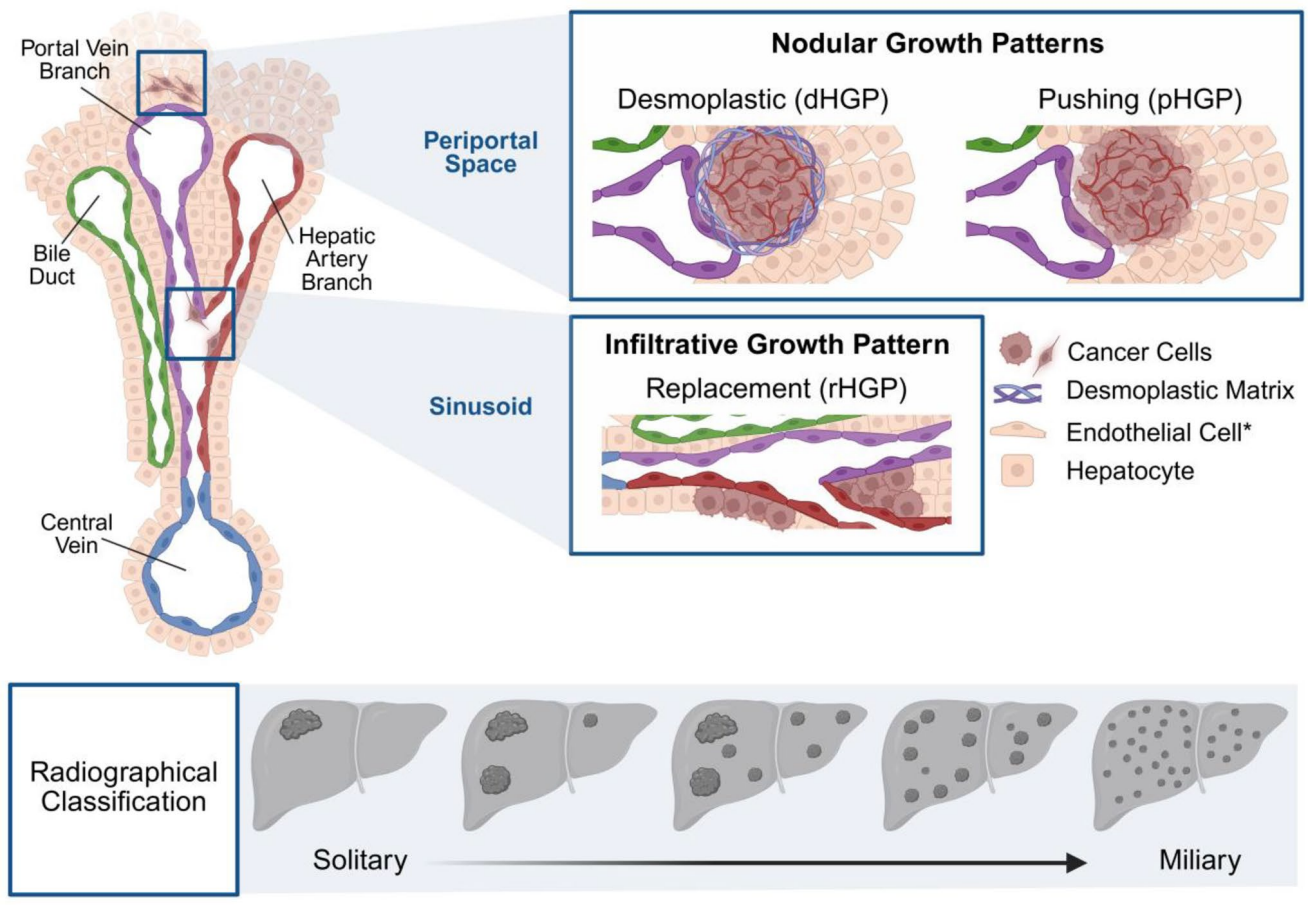
Growth patterns of LMUM. Schematic representation of the growth patterns observed in uveal melanoma liver metastases. Nodular growth patterns are characteristic of cells that seed in the periportal space with pushing (pHGP) and desmoplastic (dHGP) patterns that often damage nearby tissues. The dHGP is characterized by the distinct desmoplastic tissue and matrix surrounding the tumor that is absent in the pHGP. Infiltrative growth patterns are characteristic of cells that seed in the sinusoid, where cancer cells grow to replace existing hepatocytes without damaging the architecture of the liver (rHGP). *endothelial cells are colored based on where they exist in the hepatic triad: green (bile duct), purple (portal branch), red (hepatic artery branch). Below are images representing the radiographical classification based on the number and size of metastases present

Cells that seed in the sinusoidal space typically adopt an infiltrative growth pattern, also referred to as the replacement histological growth pattern (rHGP) (Fig. 3) [48, 49]. In this pattern, tumor cells infiltrate within the hepatocyte plates — rows of hepatocytes lining the sinusoids — gradually replacing hepatocytes while maintaining the structural framework of the liver. This allows tumor cells to co-opt existing hepatic vasculature, thereby circumventing the need for angiogenesis. Tumors with an rHGP are associated with poorer prognosis and resistance to therapy [29]. Lesions exhibiting this infiltrative pattern are generally smaller in size, often < 50 µm in diameter [48].

By contrast, nodular growth pattern lesions vary more widely in size, ranging from < 50 µm to > 500 µm, indicating distinct biological behavior and metastatic dynamics [48]. Nodular LMUM tumors arise from cells that seed the periportal space and include growth patterns such as desmoplastic (dHGP) and pushing (pHGP) (Fig. 3) [48, 49]. The dHGP is marked by a dense fibrotic rim that separates the tumor from the surrounding liver parenchyma, accompanied by neovascularization at the rim. In contrast, the pHGP lacks this desmoplastic reaction. Unlike rHGP tumors, nodular lesions exhibit stem-like and pro-angiogenic features, including elevated expression of VEGF and PEDF [48]. Interestingly, angiogenic growth patterns, similar to the dHGP tumors, respond better to anti-angiogenic therapies such as bevacizumab as seen in colorectal cancer [50].

Beyond growth patterns and histological markers, LMUM can also be classified radiologically by liver tumor burden: solitary (one lesion), oligometastatic (2–5 lesions), limited polymetastatic (6–10 lesions), extensive polymetastatic (> 10 lesions), and miliary (innumerable small lesions throughout the liver) (Fig. 3) [51]. Notably, solitary lesions more often display nodular growth, whereas miliary lesions are associated with infiltrative rHGP features [51]. Prognosis worsens with increasing lesion count, with the miliary pattern correlating with the poorest outcomes [51].

Whereas the majority of UM patients develop liver metastases within 2–5 years following primary tumor diagnosis, a subset of patients experiences extended periods of latency, with metastatic lesions manifesting 10–40 years after initial diagnosis [52, 53]. This prolonged latency is likely attributed to tumor cell dormancy, a poorly understood phenomenon that significantly impacts disease progression, treatment resistance, and overall patient mortality [12, 54]. Notably, while the presentation of liver metastases can take decades in some patients, circulating UM cells have been detected at the time of initial diagnosis, suggesting that UM intravasation occurs early in disease progression [55]. Along these lines, postmortem analysis indicates that circulating UM can successfully form micrometastases in multiple organ sites, albeit with liver micrometastases found at the highest density [56]. The mechanisms underlying both the maintenance and emergence from dormancy have been difficult to pinpoint. However, recent work, including altered neddylation pathway [57], reduced activity of ribosomal S6 kinase [58], and adiponectin levels [51] may provide some insights into this phenomenon. Interestingly, latent and extrahepatic metastases are more frequently seen in MUM tumors with mutations in the splicing factor gene *SF3B1* (splicing factor B3 subunit 1), with metastasis occurring a median of 8.2 years after the primary diagnosis [59]. *SF3B1* mutations result in an aberrant splicing pattern of important target genes, which could result in increased neoantigen presentation and potential therapeutic strategies [60, 61]. The etiology behind the development of latent metastases with *SF3B1* mutations is not well understood.

While only ~ 19% of MUM tumors harbor an *SF3B1* mutation, 80% of LMUM lesions harbor a deletion or inactivation in BRCA1-associated protein 1 (*BAP1*), located on chromosome 3p21 [2, 62, 63]. This high frequency of *BAP1* alterations in liver metastases suggests that *BAP1* loss may confer a selective advantage for UM cells to survive and proliferate in the liver microenvironment. While mutations in *GNAQ* and *GNA11* are considered the initiating events in UM development, “secondary” *BAP1* mutations play a critical role in disease progression and metastasis. Moreover, it is worth noting that although *SF3B1*, *EIF1AX*, and *SRSF2* are also second oncogenic events of UM, loss of function of *BAP1* is most often associated with liver metastases [10]. In primary UM, *BAP1* mutations are associated with a higher risk of metastasis and are strongly correlated with reduced overall survival [62]. BAP1 is a nuclear deubiquitinase that exerts its tumor-suppressing functions by forming deubiquitinating complexes that modulate epigenetics, cell cycle regulation, DNA damage repair, metabolism, and apoptosis [64]. Binding partners of BAP1 include O-linked N-acetylglucosamine transferase (OGT), polycomb group proteins, ASXL1/2, and host cell factor 1 (HCF1) [65]. Germline *BAP1* mutations are attributed to an inherited genetic disorder known as BAP1 tumor predisposition syndrome (BAP1-TPDS) [66]. BAP1-TPDS is implicated in a myriad of diseases, such as mesothelioma, breast cancer, clear cell renal cell carcinoma, and cutaneous melanoma [67]. In UM patients with BAP1-TPDS, tumors typically manifest at a younger age and have reduced overall survival compared to UM patients with sporadic *BAP1* mutations [68, 69]. Notably, sporadic mutations in *BAP1* can occur in UM between 6 months and 5 years of primary tumor initiation (within 2 doublings of the first UM clone) — oftentimes before diagnosis. The timing of sporadic *BAP1* mutation coincides with the onset of micrometastatic seeding [69]. While the canonical functions of BAP1 are well established, the specific molecular mechanisms by which *BAP1* mutations contribute to UM liver metastasis remain poorly understood.

## Current therapeutic avenues for MUM

**Fig. 4:**
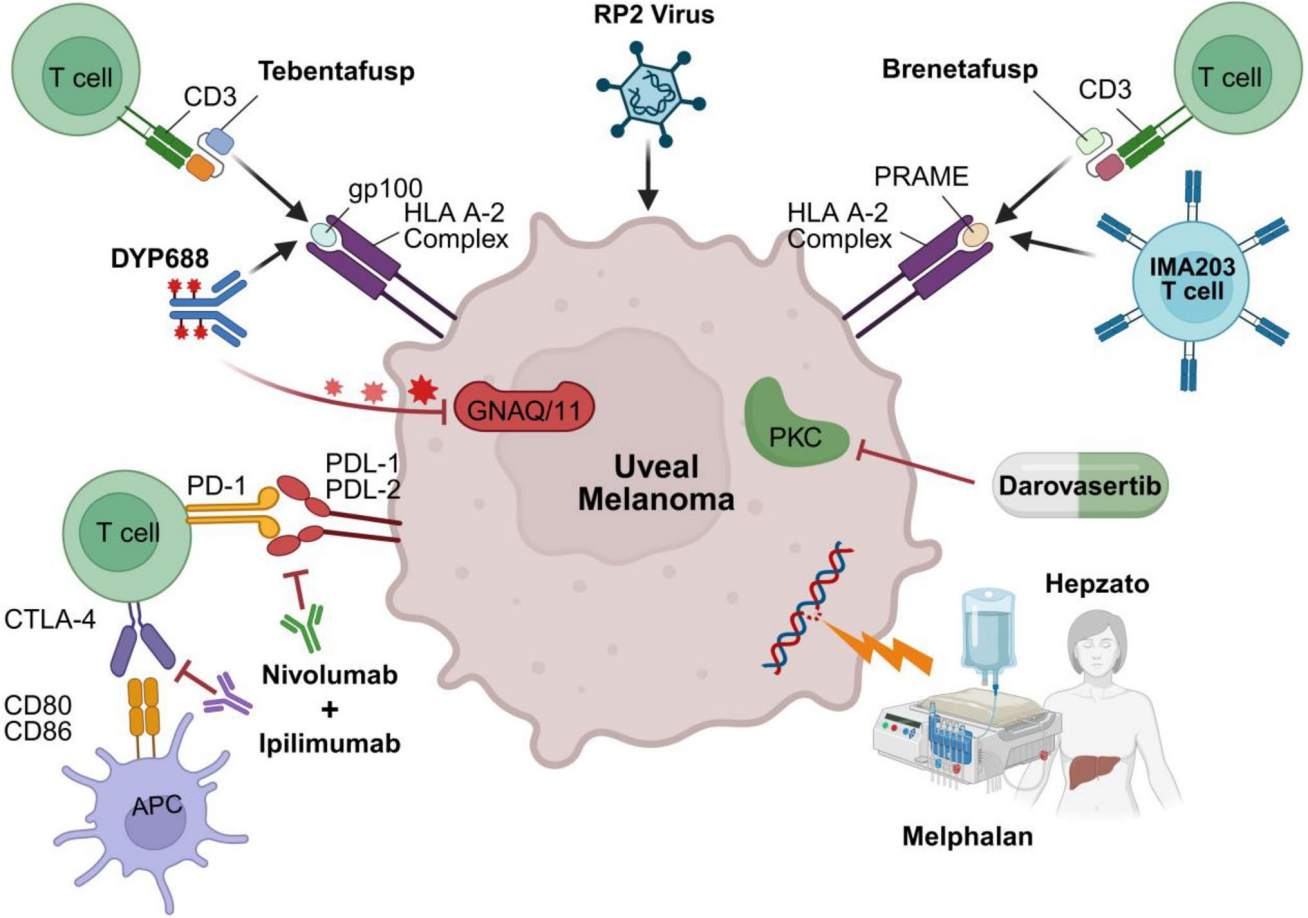
Current therapies targeting MUM. Schematic representation of FDA-approved treatments and investigational therapies for MUM. Tebentafusp/Brenetafusp function as bispecific T-cell engagers, recognizing gp100/PRAME, respectively, presented on HLA-A*02:01 complex. IMA203 is an autologous T cell product with an engineered TCR targeting PRAME presented by HLA-A*02:01. Hepzato Kit facilitates percutaneous hepatic perfusion of melphalan. Antibody–drug conjugate (ADC) DYP688 recognizes gp100, leading to the release of SDZ475, an inhibitor of GNAQ/11. Targeted therapies include darovasertib. Immune checkpoint inhibition strategies include combination ipilimab (anti-CTLA-4) with nivolumab (anti-PD-1). An oncolytic herpes simplex virus type 1, RP2, containing a codon-optimized sequence for human GM-CS, GALV-GP-R, and an anti-CTLA4 antibody-like molecule

Despite recent advances in therapies for patients suffering from MUM (Fig. 4), overall prognosis remains poor. Patients who undergo surgery (resection or ablation) have a significantly prolonged overall survival (OS), but surgical intervention is only an option for a small subset of patients with oligometastatic liver metastases [70–76]. Furthermore, surgical resection is aborted in about 30% of patients due to intraoperatively unresectable disease [77] and unfortunately, despite successful surgical resection, patients can experience relapse/disease progression potentially due to pre-existing dormant micro-metastasis commonly observed in LMUM [74].

While immune checkpoint inhibition (ICI) has significantly improved outcomes for metastatic cutaneous melanoma patients, it has been less efficacious for MUM. UM tumors have a low mutational burden, which is thought to contribute to their low immunogenicity and poor responses to immunotherapy [60, 78]. Objective response rates (ORR) are low in MUM, ranging from 5% with ipilimumab (CTLA-4 inhibitor), 3.6% with anti-programmed cell death 1 (PD-1) antibodies, 10–21% with combination ipilimumab and nivolumab (ipi/nivo), and 7.7% with combination relatlimab and nivolumab [79–88]. Interestingly, ipi/nivo was found to yield a better ORR and increased survival for MUM patients *without* hepatic metastases [89] — an effect which may be due to poor immunoreactivity found in LMUM lesions [90]. Indeed, LMUM has been shown to manifest an immunosuppressive microenvironment through mechanisms including augmented expression of the kynurenine-producing enzyme TDO [91], reduced PD-L1 and PD-L2 levels [92], and limiting dendritic cell activation [93]. In addition, it has been shown that LMUM TIL have elevated levels of the exhaustion marker LAG3 [92].

To improve MUM patient survival, efforts have been aimed towards developing novel therapeutic approaches. Tebentafusp was approved by the FDA in 2022 for the treatment of HLA-A*02:01-positive patients with metastatic UM. Tebentafusp is a bispecific T cell engager that binds to two different targets: the gp100 peptide found on uveal melanoma cells and T cell-derived CD3 [94, 95]. It is designed to recruit and activate T cells towards the gp100 peptide-HLA complex on UM cells. In the randomized phase 3 trial comparing tebentafusp to investigator’s choice (pembrolizumab, ipilimumab, or dacarbazine), tebentafusp improved OS by ~ 6 months and reduced the relative risk of death by 49% [94]. At 1 year, patients treated with tebentafusp had 73% OS compared to 59% in the control group [94]. The ORR was 11% with tebentafusp compared to 5% with the control arm. While tebentafusp is a breakthrough therapy for patients suffering from MUM, this bispecific T cell engager only targets gp100 that is presented by HLA-A*02:01, a subtype that is expressed in approximately 50% of Caucasians and represents ~ 45% of the total UM patient pool. Thus, the use of tebentafusp is limited to a subset of eligible LMUM patients [94, 96]. Moreover, tebentafusp efficacy wanes as the size of liver metastases increases, as patients with the largest lesions (> 3 cm) had 1-, 2-, and 3-year survival rates of just 52%, 26%, and 15%, respectively [97].

Similar to tebentafusp but with a different peptide target, a bispecific T cell engager that binds to the preferentially expressed antigen in melanoma (PRAME) peptide on UM cells has also been developed, called IMC-F106C, or brenetafusp. Brenetafusp also requires HLA-A*02:01 positivity. Frequent PRAME mRNA expression is well documented in cutaneous and ocular melanomas [98]. PRAME expression is considered to be a biomarker for predicting metastasis in DecisionDx Class 1 and Class 2 UM patients [99]. A PRAME-positive result may indicate an increase in metastatic risk for a Class 1 UM and a shorter time to metastasis for Class 2 UM. Class 1 patients expressing PRAME have a 38% chance of metastasis, while those defined as PRAME negative are not expected to develop metastatic disease. Moreover, patients classified as Class 2 and PRAME + have a 71% chance of metastasis within 5 years [99]. In the phase 1 trial of brenetafusp, three of six patients (50%) with MUM who were tebentafusp-naive achieved a PR [100]. None of the five patients with MUM who previously received tebentafusp responded to therapy [100]. Furthermore, brenetafusp has an improved half-life of three weeks (NCT04262466) compared to the 7.5-h half-life of tebentafusp, which could lead to improved outcomes for targeting PRAME compared to GP100 [101].

Alternative immunotherapeutic efforts have been made in developing autologous T cell therapies for UM. A phase 2 trial of adoptive transfer of autologous tumor-infiltrating lymphocytes (TIL) in LMUM patients experienced a 35% overall response rate with six partial responses and one complete hepatic response in a small phase 2 trial of 20 patients (NCT01814046) [102]. Apart from TIL therapies, efforts have been made to develop an engineered T cell therapy for patients with tumors expressing PRAME, given the high expression of PRAME in UM and known association with metastasis. An autologous T cell therapy with an engineered T cell receptor (TCR) targeting PRAME-derived peptide presented by HLA-A*02:01, IMA203, has been recently designed [103]. Notably, a phase 1 trial was recently completed and demonstrated strong responses across many tumor types, with durable response against melanoma subtypes [103]. Although only 3 MUM patients were enrolled in this trial, two of the three patients had at least a 60% reduction in lesion diameter. Importantly, the phase 1 trial also demonstrated IMA203 T cell infiltration and clinical response in heavily pre-treated patients — including UM patients — suggesting that this T cell product may be efficacious against immunologically “cold” UM liver metastases [103]. Larger-scale testing of IMA203 in MUM patients is warranted.

Another novel immunotherapeutic approach is the use of RP2, an oncolytic herpes simplex virus type 1 that contains a codon-optimized sequence for human granulocyte–macrophage colony-stimulating factor (GM-CSF), the gibbon ape leukemia virus surface glycoprotein with the R sequence deleted (GALV-GP-R-), and an anti-cytotoxic T lymphocyte antigen 4 (CTLA-4) antibody-like molecule. These modifications are intended to increase oncolytic potency, cell-to-cell spread, tumor antigen release, and systemic anti-tumor immune activation. In a phase 1 study evaluating intratumoral injections of RP2 with and without nivolumab in previously treated MUM patients, ORR was 28.6% (4/14) with a disease control rate of 57.1% (8/14) [104]. Median duration of response was 5.8 months. A randomized phase 2/3 clinical trial of RP2 plus nivolumab vs ipi/nivo in immune checkpoint inhibitor-naïve patients with MUM is ongoing (NCT06581406). While results for RP2 are preliminary and do not differentiate between hepatic and extra-hepatic metastasis, oncolytic viruses have been studied in other liver metastatic cancers. Specifically in colorectal cancer, *in vivo* and clinical trial results indicate promising effects — reducing hepatic metastatic tumor burden [105, 106].

In addition to tebentafusp, the FDA recently approved the Hepzato kit (melphalan for injection/hepatic delivery system) on August 14, 2023, for unresectable LMUM affecting less than 50% of the liver and no extrahepatic disease, or extrahepatic disease limited to the bone, lymph nodes, subcutaneous tissues, or lung that is amenable to resection or radiation. The percutaneous hepatic perfusion (PHP) procedure uses double-balloon catheters in the inferior vena cava to temporarily isolate the hepatic venous outflow. High doses of melphalan, a DNA alkylating chemotherapeutic, are then administered via an infusion catheter placed in the hepatic artery, followed by extracorporeal filtration to reduce systemic exposure to melphalan. Treatment is repeated every 6 to 8 weeks for up to 6 treatment cycles. In the phase 3 FOCUS trial, PHP resulted in an ORR of 36.3% (*n* = 33), with ~ 9 months progression-free survival (PFS), including 7.7% of patients experiencing complete response [107]. Median OS was 20.5 months, with a 1-year OS of 80% [107].

Notably, while hepatic UM was effectively treated with liver-targeted high-dose melphalan, many of these patients unfortunately progressed with extrahepatic metastases [108], suggesting that a systemic treatment may be needed to complement the liver-targeted therapy. To this end, more complex combinatorial treatments are in development, including the addition of percutaneous hepatic melphalan perfusion with ipi/nivo [109]. In the phase 1b part of the CHOPIN trial, combination treatment resulted in an ORR of 85.7% (6/7), with 14% and 71% of patients being complete responders and partial responders, respectively [109]. The phase II part of the CHOPIN study is ongoing (NCT04283890).

In addition to the liver-directed and immunotherapy-based approaches described above, there have been numerous studies evaluating targeted therapies. Directly targeting the oncogenic drivers, *GNAQ/11*, with macrocyclic depsipeptides such as FR900359 or YM-254890 showed promise *in vitro*, but have not been evaluated for clinical application [110]. Since the leading GNAQ/11 inhibitors act on both mutant and wild-type proteins, safety/toxicity concerns are limiting clinical progress with these agents. To combat this, an antibody drug conjugate, DYP688, was developed [111]. DYP688 binds to PMEL7/GP100 on the UM cells and delivers the GNAQ/11 inhibitor, FR900359. In clinical trial, over half of the patients enrolled had hepatic metastasis and 63.6% of treated patients achieved stable disease (NCT05415072).

Apart from targeting GNAQ/11, attempts have been made to target downstream signaling molecules that are activated by *GNAQ*/*11* mutations, such as the PI3K/AKT/mTOR pathway [10]. Among downstream signaling, activating mutations in *GNAQ/11* in UM are associated with increased protein kinase C (PKC) activation and, importantly, small molecule inhibitors of PKC (PKCi) effectively blocked *in vitro* UM growth and in pre-clinical models [112, 113]. Synergistic effects have also been demonstrated preclinically when combining PKCi with MEK inhibitors (MEKi) or c-MET inhibitors [112, 114, 115]. These results spurred clinical trials investigating the therapeutic efficacy of PKCi as a monotherapy or in combination with other targeted therapies. The PKCi, darovasertib (LXS196, IDE196), is the most promising [116, 117]. In 2022, the FDA granted orphan drug status for darovasertib for the treatment of UM. A phase 1/2 clinical trial evaluated darovasertib as monotherapy or in combination with the MEK inhibitor binimetinib or MET inhibitor crizotinib (NCT03947385). Preliminary results demonstrate that darovasertib as a monotherapy had a 57% 1-year OS in the second line, third line, and heavily pre-treated MUM patients [116]. Median OS was 13.2 months, and a decrease in tumor size was seen in 46 of the 75 (61%) of MUM patients, with 15 patients experiencing a PR (20%). The preliminary results for the darovasertib and binimetinib combination showed that in 14 evaluable MUM patients, 79% experienced tumor reduction, with two of nine patients achieving PR (22%) [116]. Even more impressive was the combination of darovasertib with crizotinib, with 92% of patients achieving tumor reduction (*n* = 63) with a 30% confirmed PR [116, 118]. Median PFS was ~ 7 months. First-line MUM patients (*n* = 20) had a confirmed PR of 45%, and 95% of patients had tumor shrinkage. Deep and sustained circulating tumor DNA molecular response was seen in the majority of patients [118]. The potentially registration-enabling phase 2/3 trial evaluating the combination of darovasertib and crizotinib in the first-line setting in patients with HLA-A2-negative MUM is ongoing (NCT05987332).

The inherent chemotherapy resistance [119] and heterogenous nature of UM [120] may underpin the above clinical challenges; and while strides have been made to improve outcomes for MUM patients, further work is needed to provide meaningful, durable responses.

## Immunological landscape of LMUM

While the liver possesses one of the richest immune cell populations in the body, LMUM are immunologically “cold” tumors — often presenting with inactive or pro-tumor immune infiltrates — an effect that can limit the efficacy of many of the therapies discussed above. Furthermore, few studies have resolved the immunomodulatory characteristics of LMUM due to the scarcity of viable peritumoral samples [121, 122]. Deciphering the immune infiltrates in LMUM can enhance therapeutic avenues.

The liver’s extensive blood filtration exposes it to a constant stream of antigens, making it one of the body’s most abundant sites for resident and migratory immune cell populations—including both adaptive and innate immune cells that maintain homeostasis and regulate inflammation in response to hepatic injury and disease (reviewed in [123, 124]). Adaptive liver immune cells include CD4 +, CD8 +, γδ-T, and NKT cells. Hepatic CD4 + helper T cells (Th1, 2, 17, and Tfh cells) stimulate allergic responses, cytokine release, and B cell proliferation [125]. CD4 + derived regulatory T cells (CD4 + Tregs) are crucial to maintaining self-tolerance and immune homeostasis by interacting with innate immune cells, such as Kupffer cells (KCs), to stimulate a local immune repressive response. LMUM tumors with increased infiltration of CD4 + T Fox3P + cells, Tregs, had poor overall survival and increased resistance to immunotherapy [121]. Hepatic CD8 + T cells consist of cytotoxic T cells—the primary effector cells in adaptive immunity—and CD8 + Tregs, which inhibit Th cells and inflammatory responses [125]. CD8 + T cells do infiltrate LMUM; however, they are functionally repressed and display a dormant phenotype—low expression of cytotoxic genes [90]. In UM, high CD8 + T cell infiltrates are correlated to better response to isolated hepatic perfusion with melphalan [126], and tebentafusp, a bispecific T cell engager targeting gp100 and T cell-derived CD3, can drive infiltration of CD8 + and CD3 + T cells and gene signatures corresponding to active immune cells. γδ-T cells are another class of T cells found in the liver microenvironment and can recognize cancer cells independent of the cancer cells’ HLA, and γδ-T cells express natural killer (NK) receptors to recognize stress molecules on cancer cells [127]. In primary uveal melanoma, infiltration of γδ-T cells is associated with poor overall survival and increased metastasis [128, 129]. However, the role γδ-T cells play in the survival and growth of UM in the liver has yet to be uncovered. In hepatic cancers and hepatic metastasis, γδ-T cells can display an anti-tumor effect and may play a similar role in LMUM [129]. NKT cells are predominantly found in the liver and are involved in tumor rejection, immune surveillance, and recognition of infection, such as viral and microbial infections [130–132]. NKT cells are classified as either Type I, anti-tumor, or Type II, immunosuppressive, in the context of cancer [133]. Pre-clinical models indicate that type I NKT cells can enhance liver metastasis after intraocular seeding and inhibit NK cell cytotoxicity; however, it is important to note that specific characteristics that may be present in uveal melanoma cells could be lacking as the cell line used in this study is B16 melanoma cells [134].

The liver contains a large pool of macrophage-like cells, indicating a critical function in hepatic homeostasis. While there are several macrophage subsets in the liver, the most prevalent are Kupffer cells (KC) and monocyte-derived macrophages [135]. Activated macrophages, including KCs, can polarize into an M1- or M2-like phenotype. M1 polarization is associated with a degenerative, pro-inflammatory phenotype, while M2 polarization is associated with a protective, anti-inflammatory phenotype (reviewed in [136]). The plasticity of liver macrophages is essential for regulating liver inflammation [137]. A recent study investigating the spatial distribution and density of immune cell populations in LMUM lesions highlights an enrichment of CD68 + and CD163 + M2-polarized tumor-associated macrophages (TAMs) — a cell type often associated with supporting a pro-tumor microenvironment [138]. Furthermore, patient sample analysis highlights how CD163 + M2-like TAMs localize close to UM cells; an effect that may limit T cell-mediated cytotoxicity [121]. Additionally, tumors that displayed a desmoplastic growth pattern had more peri-tumoral CD68 + CD163 + TAMs (indicative of the M2 phenotype), whereas replacement tumors had no significant immune pattern phenotype [139]. A potential rationale for the enrichment of M2-like TAMs in LMUM [138] may be linked to the engagement of macrophage-derived SIRPα. UM have elevated CD47 [140] a cell surface receptor that binds to SIRPα — this interaction can induce M2 polarization [141] and suppresses macrophage phagocytosis, acting as a “don’t eat-me” signal. Interestingly, enhancing phagoptosis (killing of cells via phagocytosis) by inhibiting “don’t eat-me” signals has been explored in leukemia [142], breast cancer [143], multiple myeloma [144], cutaneous melanoma [145], and others [146, 147]. Inhibitory monoclonal antibodies against CD47 (Hu5F9-G4, AK117) and SIRPα (CC-95251) — intended to quench the “don’t eat-me” signal and/or suppress pro-tumor M2 polarization of TAMs, are currently in clinical trials for various cancers; however, the effects of these agents in LMUM are unknown.

The liver is also home to hepatic dendritic cells (HDC), antigen-presenting cells that can produce cytokines, uniquely migrate to the lymph node, and stimulate adaptive immunity *via* presentation of antigens [148]. Interestingly, to enhance anti-tumor immunity, UM patients with hepatic and extrahepatic metastases were enrolled in a phase 1 trial to analyze the effects of DC vaccination whereby DCs are loaded with the melanoma antigens, gp100 and tyrosinase [149, 150]. Vaccines with DCs were administered through various routes (intravenous, intradermal, or intranodal), inducing tumor-specific immune responses in 29% of patients and achieving a median OS of 19.2 months without severe toxicities [149]. Furthermore, tumors treated with DC vaccines had increased tumor-specific CD8 + T cells and NK cells [149]. More recent studies demonstrate IKKβ-matured RNA-transfected DCs can enhance both adaptive and innate immune responses when loaded with amplified tumor mRNA and personalized UM-associated antigens based on exome and transcriptome sequencing [150]. DC vaccines could be a potential therapeutic avenue to flip LMUM from immunologically cold to hot.

NK cells are a component of the innate lymphoid cells and function in immune defense mechanisms as immunosurveillance and in producing cytokines and chemokines [151–153]. Activation of NK cells through receptor-ligand binding can occur through three types of receptors: natural cytotoxicity receptors, Fc receptors, and C-type lectin receptors [154–158]. This activation results in the release of cytokines, such as IFN-γ and tumor necrosis factor (TNF), and chemokines, including CCL3, CCL4, CCL5, and CXCL8 [159–163]. Conversely, the binding of inhibitory ligands, HLA-E and HLA-C, to their cognate receptors on NK cells protects healthy cells from inappropriate targeting by the immune system [164–166]. Intriguingly, UM cells express NK cell ligands — HLA-A/B/C, HLA-E — that, when bound to the receptor on the NK cell, can have an inhibitory effect [167, 168]. Another mechanism of inhibiting NK cell activation in primary UM is by the secretion of TGF-β either by UM cells or other cells in the microenvironment, such as M2 macrophages [169, 170]. Similarly, LMUM has mechanisms to inhibit NK cells through the expression of MHC class I molecules [171, 172]. However, a more complete understanding of how NK cells affect metastasis and dormancy is needed.

Neutrophils are the predominant granulocyte in the blood and migrate and accumulate in the sinusoids of the liver in response to chemokines and chemotactic agents [173]. Neutrophils are retained in the liver through the expression of CXCR6, a G protein-coupled receptor that binds to its ligand, CXCL16, expressed on endothelial cells and macrophages in the liver [174, 175]. Neutrophils initiate clearance via phagocytosis and secrete granules containing proteolytic enzymes, MMPs, lysozymes, and bactericidal proteins [176–178]. In LMUM patients, the ratio of neutrophils to lymphocytes (NLR) can be utilized as a prognostic marker after ICI, namely ipi/nivo, with elevated NLR potentially indicating reduced ICI response [179].

## Discussion

Though recent research has provided valuable insights, the molecular mechanisms driving UM’s liver tropism remain elusive. The high frequency of *BAP1* inactivation in LMUM suggests this tumor suppressor plays a crucial role in the metastatic process [62, 180]. Considering its roles in epigenetic regulation, *BAP1* loss likely disrupts gene expression that might contribute to immune evasion and/or metabolic adaptations necessary for survival in the hepatic microenvironment; however, the precise mechanisms by which *BAP1* deficiency promotes liver-specific metastasis require further investigation [64, 181]. Recently, our group identified a role for *BAP1* loss in lipotoxicity resistance [182]. In efforts to mimic the liver microenvironment, we found that *BAP1* WT UM cells were sensitive to exogenous lipids, exhibiting hallmarks of ferroptotic-like oxidative stress. Conversely, *BAP1* mutant UM cells were resistant to lipids. We further highlight how lipid sensitivity is mediated through the BAP1/ASXL2 axis, as knockdown of ASXL2 phenocopied lipotoxicity resistance observed in *BAP1* mutant UM. Importantly, we demonstrate how disrupting lipid metabolism with atorvastatin sensitizes *BAP1* mutant UM by inducing a ferroptotic-like death in the liver microenvironment [182]. Notably, ferroptosis can be considered an inflammatory/immunogenic cell death, associated with the release of immunostimulatory factors [183]; thus, targeting lipid metabolism in LMUM may stimulate a “hot” tumor immune microenvironment, amenable to a more durable therapeutic response.

As the liver is the predominant site for UM metastasis, liver-specific microenvironmental factors might facilitate tumor cell homing, colonization, dormancy, and subsequent proliferation. The liver’s zonation and oxygen gradient may influence how LMUM manifests, potentially explaining the differences between nodular and infiltrative growth patterns and varying responses to hypoxic environments [184–186]. Furthermore, nodular lesions, with their enhanced angiogenic properties, might respond differently to therapies compared to infiltrative lesions [48]. Additionally, the liver’s zonation creates distinct niches that could underlie metabolic heterogeneity [187], and may dictate sensitivity to metabolic perturbations [182, 188–190]. In addition to continued pre-clinical dissection of LMUM vulnerabilities, improved detection and classification of distinct LMUM subgroups may facilitate more personalized therapeutic strategies and improve treatment outcomes.

**Fig. 5:**
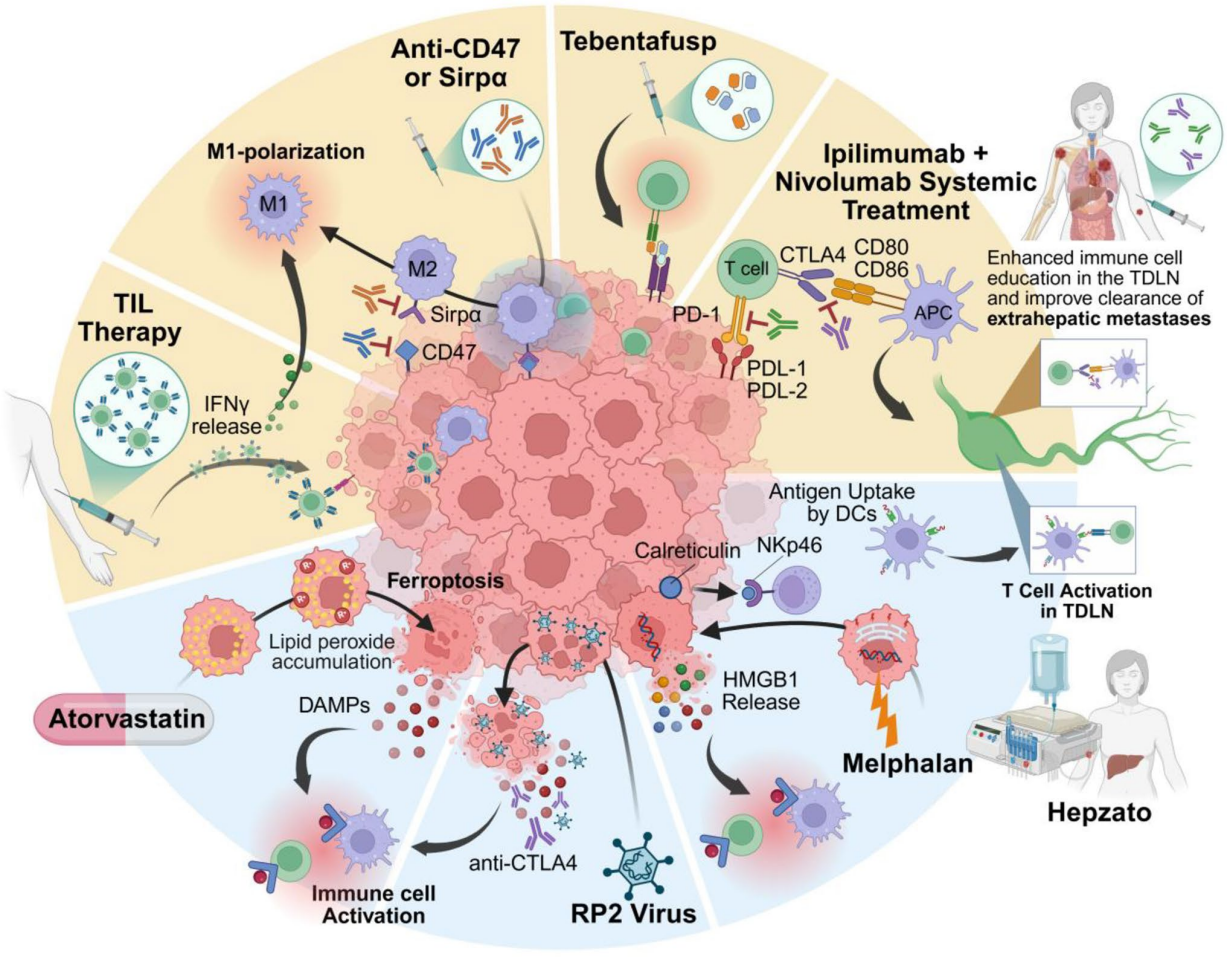
Prospective multimodal theraputic strategies for targeting LMUM. Overview of immunotherapy strategies and their potential infuence on the LMUM microenvironment. Yellow zones indicate therapies intended to reprogram immune cells within the microenvironment. Blue zones contain therapies capable of inducing immunogenic or inflammatory cell death. Combining therapies in each of these zones has the potential to produce synergistic tumor clerance effects and bolster immune response

Similar to other liver metastatic cancers — colorectal, breast, and cutaneous melanoma — LMUM has an immunogenic “cold” phenotype. This immunological “cold” nature of LMUM presents perhaps the most significant therapeutic challenge [94, 121, 122]. Unlike other immunogenic tumors, LMUM demonstrates remarkable resistance to immune checkpoint inhibition [90]. This resistance might stem from the liver’s specialized immune cell landscape. Of note, most liver metastatic cancers have M2-infiltrating macrophages [139, 191, 192] which indicate an inherent predisposition for an immune-cold tumor phenotype in the liver microenvironment. The liver also contains a significant population of resident macrophages in the body, KCs, which can exhibit plasticity in their functional phenotypes [193]. LMUM exploits the plasticity of the macrophages through the CD47-Sirpα axis, altering the immune microenvironment and decreasing T cell response (Fig. 5) [140]. Additionally, the liver harbors a unique composition of lymphocytes, with an enrichment of innate lymphoid cells and NK cells [194, 195]. Similarly to cutaneous melanoma, immunotherapies, ipi/nivo, have been used to treat metastatic uveal melanoma. However, patients without hepatic metastasis had better ORR than patients with hepatic metastasis, indicating that ipi/nivo alone is not enough for LMUM [89]. In response to these immunological challenges, significant advances in novel therapeutic strategies have emerged. Current clinical trials are investigating the efficacy of tumor-infiltrating lymphocyte (TIL) therapy and have had modest effects in the treatment of LMUM (NCT01814046) [102]. Other strategies focus on recruiting T cells to an immunologically cold tumor and facilitating anti-UM cytotoxicity, including Tebentafusp, brenetafusp, and IMA203 [94, 114–116, 196].

Apart from immunotherapies, efforts have been made towards therapeutics that could elicit an immune response, such as Hepzato Kit — a liver-directed percutaneous hepatic perfusion that directly delivers melphalan to the liver. *In vivo* models of B-cell lymphoma and colorectal cancer demonstrated that melphalan causes immunogenic cell death, determined by surface-exposed calreticulin and release of high-mobility group box 1 (HMGB1) [197]. Additionally, melphalan treatment enhanced tumor antigen uptake by dendritic cells in tumor-draining lymph nodes (TDLN) and led to activation of CD8 + T cells [197]. In cutaneous melanoma, melphalan treatment was associated with enhanced expression of the pro-inflammatory cytokines: IL-1β, IL-8, and IL-6 and was found to induce apoptosis through ER stress and reactive oxygen species [198]. While these studies were not UM models, Hepzato may have similar effects and stimulate anti-UM immunity. Furthermore, the CHOPIN study, which combined Hepzato with Ipi/Nivo, is encouraging [109]; these results may be attributed to the immunostimulatory effects of melphalan working in concert with Ipi/Nivo. Potential clinical avenues could examine the therapeutic potential of a combination therapy composed of agents that could induce immunogenic/inflammatory cell death, atorvastatin or the oncolytic virus RP2, with therapies to counteract the immunosuppressive nature of LMUM — such as Ipi/Nivo, anti-CD47, or Tebentafusp (Fig. 5**)**. Together, these therapeutics may recruit T cells and enhance anti-tumor immune responses in LMUM.

The selective tropism of UM metastases for the liver, coupled with the poor prognosis and limited treatment options for MUM patients, highlights the critical need for a deeper understanding of the interactions between metastatic UM cells and the specialized hepatic microenvironment. Additionally, therapeutic strategies could be improved with better preclinical models, as current studies often rely on cell lines from primary rather than metastatic lesions. Developing murine syngeneic models could provide more accurate insights into therapeutic response and durability. Advancing sophisticated experimental systems, identifying predictive biomarkers, and elucidating mechanisms of liver tropism will be crucial for developing more effective treatments and extending patient survival.

## Data Availability

No datasets were generated or analysed during the current study.
